# How energy balance-related behaviours, temperament, stress and overweight associate: a cross-sectional study of Finnish preschoolers

**DOI:** 10.1017/S1368980024000612

**Published:** 2024-03-04

**Authors:** Henna Vepsäläinen, Liisa Korkalo, Essi Skaffari, Anna M Abdollahi, Riikka Pajulahti, Reetta Lehto, Elina Engberg, Marja H Leppänen, Elviira Lehto, Carola Ray, Eva Roos, Maijaliisa Erkkola

**Affiliations:** 1 Department of Food and Nutrition, University of Helsinki, Helsinki, Finland; 2 Folkhälsan Research Center, Helsinki, Finland; 3 Department of Psychology and Logopedics, University of Helsinki, Helsinki, Finland; 4 Department of Public Health, Clinicum, University of Helsinki, Helsinki, Finland; 5 Department of Food Studies, Nutrition and Dietetics, University of Uppsala, Uppsala, Sweden

**Keywords:** Lifestyles, Obesity, Dietary intake, Early childhood, Long-term stress, Extraversion, Finland

## Abstract

**Objective:**

This study aimed to (1) examine the clustering of energy balance-related behaviours (EBRB) and (2) investigate whether EBRB clusters, temperament and hair cortisol concentration (HCC) associate with overweight.

**Design:**

We assessed food consumption using food records, screen time (ST) using sedentary behaviour diaries, sleep consistency and temperament (negative affectivity, surgency, effortful control) using questionnaires and HCC using hair samples. Accelerometers were used to assess physical activity (PA) intensities, sleep duration and sleep efficiency. Researchers measured each child’s weight and height. We used finite mixture models to identify EBRB clusters and multilevel logistic regression models to examine the associations between EBRB clusters, temperament, HCC and overweight.

**Setting:**

The cross-sectional DAGIS survey, data collected in 2015–2016.

**Participants:**

Finnish 3–6-year-olds (*n* 864) recruited through preschools.

**Results:**

One-third of the participants were categorised into the cluster labelled ‘Unhealthy diet, excessive screen time’, characterised by unhealthy dietary choices (e.g. greater consumption of high-fat, high-sugar dairy products) and longer ST. Two-thirds were categorised into the second cluster, labelled ‘Healthy diet, moderate screen time’. PA and sleep were irrelevant for clustering. Higher negative affectivity and lower effortful control associated with the ‘Unhealthy diet, excessive screen time’ cluster. EBRB clusters and HCC did not associate with overweight, but surgency was positively associated with overweight (OR = 1·63, 95 % CI 1·17, 2·25).

**Conclusions:**

Of the EBRB, food consumption and ST seem to associate. As temperament associates with EBRB clusters and overweight, tailored support acknowledging the child’s temperament could be profitable in maintaining a healthy weight.

Societies have been unable to find solutions to the obesity crisis despite the seemingly simple cause for overweight (including obesity) – the imbalance between energy intake and expenditure^(1)^. Mounting evidence suggests that food systems and an increasingly obesogenic environment are the main drivers of the obesity epidemic^(2)^, but more knowledge on the associations of individual characteristics, behaviours and overweight is needed to better understand how individuals behave in their environments. The so-called energy balance-related behaviours (EBRB), such as food consumption, physical activity (PA) and sedentariness, are proximally associated with energy intake and expenditure, and they may thus directly impact overweight already in childhood. Studies have found associations between, for example, low levels of sedentary behaviour and TV viewing, high levels of moderate to vigorous PA and infrequent consumption of sugar-sweetened beverages with a lower BMI or a higher fat-free mass index among children^(3–6)^. However, as EBRB tend to intertwine, rather than analysing single EBRB, many studies have shifted their focus on EBRB clustering and the associations between EBRB clusters and overweight^(7–13)^.

Rather consistent evidence shows that unhealthy EBRB, such as low consumption of fruit and vegetables, high consumption of energy-dense foods, low levels of PA and high levels of screen time (ST) or other sedentary behaviours, tend to cluster among preschool-age children^(7–12)^. Similarly, healthy EBRB (e.g. varied food intake, high consumption of fruit and vegetables, low consumption of sugar-sweetened beverages, high levels of PA or low levels of ST or other sedentary behaviours) seem to interrelate^(8,9,11,12)^. Furthermore, most studies have also found associations between the unhealthy EBRB clusters and increased likelihoods of overweight, obesity or higher body fat percentage^(7,9,11,13)^. However, most published studies have assessed PA using parent-reported questionnaires and have been unable to separate between various PA intensities, which may relate differently with other EBRB. In addition, these studies have predominantly not considered dietary quality but instead use food consumption frequencies or food intake (g/d) as input variables. Thus, the clusters reported in literature are generally more descriptive of consumption frequencies or quantities of foods eaten than of dietary quality, and conclusions cannot be made of whether the link between the clusters and overweight is purely driven by energy imbalance (excess energy intake or low energy expenditure) or whether dietary quality also plays a role.

Sleep patterns, along with dietary behaviour, PA and ST, have been shown to associate both with each other and overweight^(14–16)^. Despite accumulating knowledge concerning the associations between sleep and other EBRB, sleep-related variables have not routinely been included in EBRB clustering studies^(17)^: using parent-reported questionnaires, only two studies have suggested that consistent wake-up times and bedtimes and longer sleep duration cluster with other healthy EBRB among European preschoolers^(7,12)^. However, as parents might over-estimate preschoolers’ sleep duration^(18)^, there is a need for objective sleep assessment methods. Furthermore, sleep researchers have suggested looking beyond sleep duration and also considering variables related to sleep quality, where possible^(19)^.

Multiple individual- or family-level factors, such as the socio-economic status (SES) of families, personality traits or temperament and exposure to stress hormones, may also be involved in childhood overweight^(20,21)^. For instance, poor self-regulation (the ability to monitor and manage emotions and behaviours) and low negative affectivity (the tendency to experience negative emotions) have been shown to associate with higher BMI in preschoolers^(20)^. Higher surgency (a temperament dimension marked by cheerfulness, responsiveness, spontaneity and sociability) or impulsivity have, in turn, been found to associate with greater weight gain rate in infancy^(20)^ and higher BMI in early adolescence^(22)^. However, higher surgency has also been associated with healthier EBRB (low sedentariness and more likely vegetable consumption)^(23,24)^, which emphasises the complexity of the relationship between temperament, EBRB and overweight. To further complicate the big picture, evidence indicates that long-term stress, as measured with hair cortisol concentration (HCC), may link to higher BMI^(25)^ and to a less ‘health-conscious’ dietary pattern among preschoolers^(26)^.

Taken together, it seems that EBRB cluster already in early childhood, but it remains unknown, whether the clusters and their associations with overweight are driven by food energy contents or dietary quality. In addition, there is a lack of studies incorporating objective and versatile measurements of sleep and PA in EBRB clusters. Furthermore, we aimed to consider temperament and HCC as individual-level factors, which may associate with both EBRB clusters and overweight. Thus, this article aimed to examine EBRB clustering – food consumption, various PA intensities, ST and sleep – within a sample of Finnish preschoolers using assessment methods that are as accurate and objective as possible (food records, accelerometers) and taking energy intake into account. We also studied whether socio-demographic factors, temperament, HCC and weight status differed between EBRB clusters. Moreover, we investigated how EBRB clusters, temperament and HCC associate with overweight. Due to the exploratory nature of the data-driven analyses, we had no a priori hypothesis for the clustering of EBRB. However, we hypothesised that surgency and HCC would be positively associated with overweight. Figure 1 illustrates the conceptual framework of the study.

**Fig. 1:**
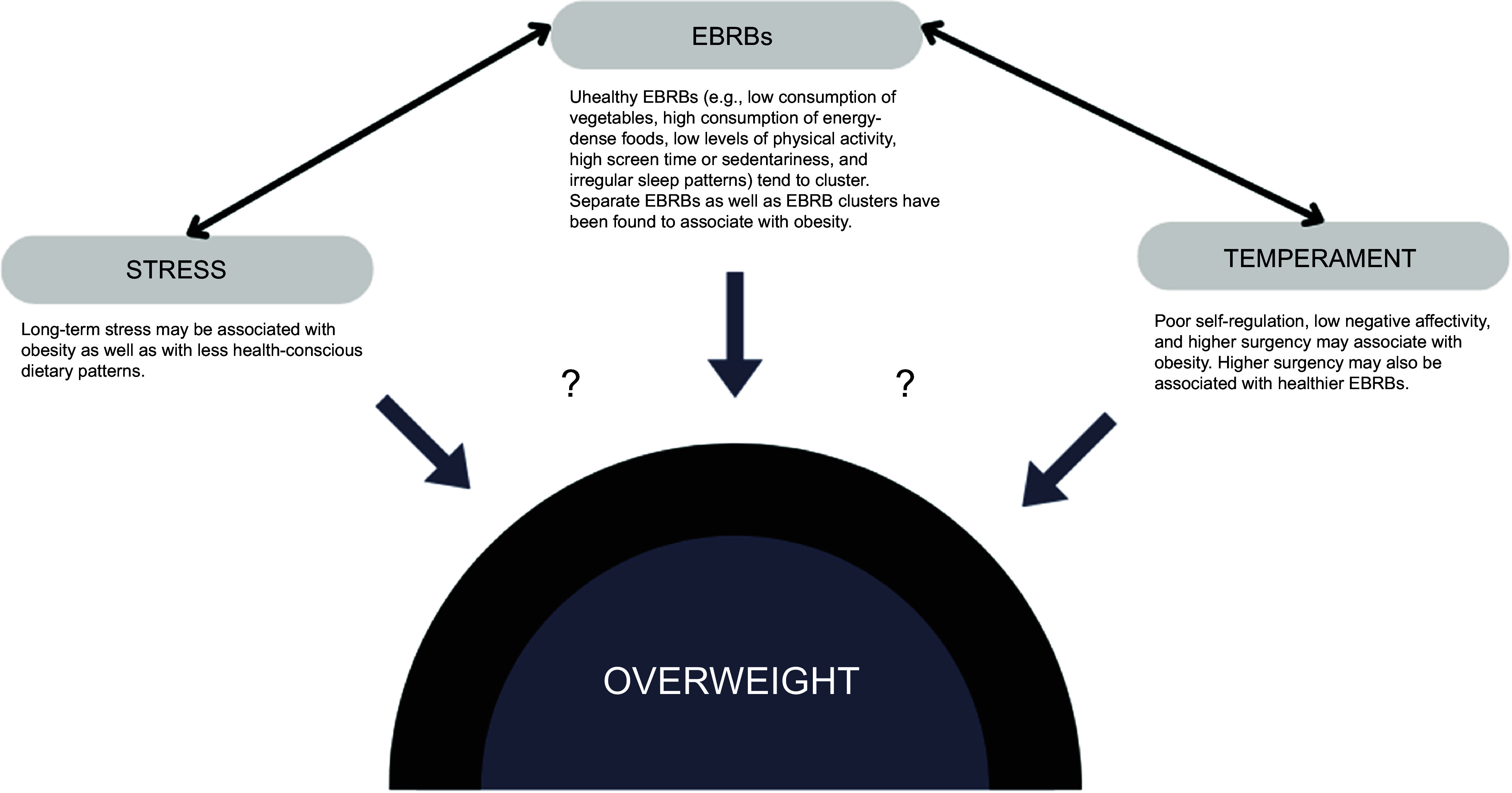
The conceptual framework of the study shows the examined associations between energy balance-related behaviours (EBRB), stress, temperament and overweight. Double-arrows refer to hypothesised bidirectional associations; question marks indicate the associations investigated

## Methods

### Study design and population

This article reports secondary analyses from the DAGIS (Increased Health and Wellbeing in Preschools, www.dagis.fi) research project examining health behaviours and related factors among Finnish 3–6-year-olds. We used data from a cross-sectional survey conducted between September 2015 and April 2016 in eight Finnish municipalities. Of all municipal preschools and preschools from whom the municipalities purchased education services, we randomly invited 169 preschools to participate in the study. Of the invited preschools, sixty-seven (40 %) did not wish to participate and sixteen (9 %) were excluded due to being a 24-h preschool, operating in a language other than Finnish or Swedish (the official languages of Finland) or not offering reduced fees for low-income families. Thus, eighty-six preschools (51 % of those invited) provided written informed consent.

From the consenting preschools, we invited all 3–6-year-olds (*n* 3592) and their families to participate in the study. Due to the researcher burden of extensive measurements and limited resources, we excluded children from preschools with low participation rates (≤ 30 % participation in all eligible preschool groups; ninety-one children in twenty preschools). The legal guardians (later referred to as parents) of 892 children from sixty-six preschools provided written informed consent, and data were obtained from 864 children (24 % of those invited in total; 29 % of those invited from the participating sixty-six preschools). The power calculation for the primary data collection^(27)^ as well as the flow of the preschools and participants has previously been illustrated in detail^(28)^ (The University of Helsinki Review Board in the Humanities and Social and Behavioral Sciences reviewed the study in February 2015 (Statement 6/2015).

### Measures

#### Weight status

Trained researchers measured each child’s weight and height without shoes and heavy clothing at the preschool using portable bench scales (CAS PB-100/200). Stadiometers (SECA 217) were used to measure height. BMI (kg/m^2^) was calculated as body weight (kg)/height^2^ (m) and BMI standard deviation score (BMI-SDS) using the national references^(29)^. We used cut-offs based on the age- and sex-specific Finnish growth reference^(29)^ to separate between participants with overweight (including obesity) and participants with normal weight or thinness. To allow international comparison, we also present the results using cut-offs set by the International Obesity Task Force (IOTF)^(30)^ in the online supplementary material.

#### Energy balance-related behaviours

##### Food consumption

A 3-d food record including two weekdays and one weekend day was used to assess food consumption at home. A separate, pre-coded 2-d food record was kept at the preschool by the preschool personnel for the same two weekdays. To capture seasonal variation in diet, the families who agreed to be contacted for additional data collection were asked to fill out an additional 2-d food record 4–11 months later (between June 2016 and September 2016). Altogether 292 children (34 % of participants) provided these additional data, and the food consumption data thus consisted of 1–5 food record days for those participants and 1–3 for other participants. Both parents and preschool personnel were provided with a validated Children’s Food Picture Book^(31)^ to assist in portion size estimation, and they were instructed to record all foods eaten. The data were entered using the AivoDiet dietary software including the Fineli Food Composition Database Release 16 (2013) of the National Institute for Health and Welfare. After extracting the data from the software, each food item or mixed dish was assigned to one of ninety-seven detailed food groups. For purposes of our current study, the detailed food groups were further aggregated into twenty-eight food groups. One food group (other drinks; including water, coffee and tea) was excluded from the analyses due to inconsistent reporting in water consumption and minimal significance of coffee and tea in this age group. For each participant, daily mean consumption (g/d) of the food groups was calculated. As we were especially interested in examining the role of dietary quality in EBRB clusters and because energy intake correlates with PA, we adjusted the consumption of the remaining twenty-seven food groups with daily energy intake, and the resulting continuous variables (g/MJ) were used in the analyses (listed in Table 1).

**Table 1:** Mean values of variables considered for clustering among all participants and in the two clusters

	All participants		Cluster 1 (*n* 304): Unhealthy diet, excessive screen time		Cluster 2 (*n* 560): Healthy diet, moderate screen time		*P* value for significance
	Mean	s d	Mean	sd	Mean	sd	
Food consumption (g/MJ)							
Fresh vegetables	11·40	7·99	10·64	7·93	11·83	8·01	0·04
Vegetable dishes	5·98	7·32	5·25	6·94	6·40	7·51	0·03
Fruit and berries	20·69	14·36	20·08	15·23	21·05	13·84	0·35
Fruit soups and smoothies	6·23	9·35	6·01	9·50	6·36	9·26	0·60
Potatoes	10·19	7·48	9·71	7·47	10·47	7·48	0·16
Rye bread*	3·92	2·98	3·38	2·69	4·23	3·10	<0·01
Multigrain bread and other cereals	4·44	3·54	4·39	3·47	4·47	3·58	0·76
White bread and refined cereals	2·29	2·31	2·45	2·39	2·19	2·27	0·12
Porridge*	19·11	16·45	14·41	13·11	21·84	17·54	<0·01
Savoury pastries and pizza*	3·15	4·48	4·13	5·82	2·58	3·34	<0·01
Sweet pastries*	4·60	4·56	5·63	5·64	4·01	3·68	<0·01
Pasta and rice	5·12	5·59	5·07	6·03	5·15	5·33	0·85
Margarine	1·86	1·33	1·75	1·26	1·92	1·356	0·08
Butter and butter-fat blends	0·78	0·96	0·76	0·92	0·79	0·99	0·66
Fish and fish dishes	5·75	6·96	5·37	7·54	5·96	6·60	0·25
Red meat, meat dishes, sausages and eggs	21·38	11·94	21·39	12·52	21·38	11·61	0·99
Poultry and poultry dishes*	5·34	6·40	4·08	4·54	6·07	7·17	<0·01
Skimmed milk*	41·09	34·49	28·88	26·01	48·16	36·77	<0·01
Milk with 0·1 % or more fat*	27·50	30·65	23·33	25·01	29·92	33·27	<0·01
Flavoured milk and ice cream*	5·73	9·62	10·30	13·80	3·08	4·10	<0·01
Yogurt	10·81	11·62	9·52	11·63	11·56	11·56	0·02
Cheese	1·98	2·04	1·79	2·05	2·10	2·03	0·04
Other milk and plant-based products*,†	8·53	17·94	16·72	27·00	3·79	5·02	<0·01
Sweets and chocolate	2·02	2·58	2·01	2·66	2·03	2·54	0·90
Jams and sugar*	0·81	1·22	1·02	1·56	0·69	0·95	<0·01
Drinks*,‡	17·00	16·53	21·19	19·70	14·58	13·84	<0·01
Other foods*,§	2·41	2·42	2·96	3·13	2·09	1·83	<0·01
Screen time (min/day)							
TV and DVD watching*	59·46	31·44	58·45	36·72	60·05	27·94	0·51
Computer, tablet and smart phone use*	17·89	24·13	33·67	31·87	8·63	9·86	<0·01
Physical activity (min/h)							
Sedentariness	29·76	3·25	29·77	3·41	29·75	3·16	0·95
Light physical activity	24·81	2·44	24·75	2·56	24·84	2·37	0·65
Moderate physical activity	3·92	1·07	3·96	1·11	3·90	1·05	0·45
Vigorous physical activity	1·51	0·70	1·52	0·71	1·51	0·69	0·88
Sleep							
Sleep duration (h)	9·72	0·53	9·72	0·56	9·73	0·51	0·77
Sleep efficiency	0·87	0·04	0·87	0·04	0·87	0·04	0·10
Sleep consistency||	4·26	0·62	4·25	0·60	4·27	0·63	0·72

* Variables relevant for clustering, discriminative power 0–34.34 %.

† Cream, sour milk, milk-based desserts and sauces, plant-based drinks and other plant-based alternatives.

‡ Sugared and artificially sweetened juices and soft drinks.

§ Nuts, dried fruits, other snacks, spices, spice sauces, etc.

|| Means of three parent-reported items (in the last typical week: (i) child went to bed at the same time at night; (ii) child slept the right amount; (iii) child slept about the same amount each day), range 1–5.

##### Physical activity

The participating children used a hip-worn ActiGraph wGT3X-BT accelerometer for seven consecutive days, 24 h per day. Parents reported the non-wearing time of the accelerometers (e.g. during water-based activities). We used 15-s epoch length and regarded periods of ≥ 10 min of consecutive zeroes as non-wearing time^(32)^. A valid day was defined as ≥ 600 min of awake wearing time. We used Evenson’s cut-points^(33)^ to calculate the time spent in different PA intensities. Sedentary time was calculated along with light, moderate and vigorous PA as (mean on weekdays × 5 + mean on weekend days × 2)/7 and further divided by the accelerometer wearing time and multiplied by 60 for all children who had accelerometer data from at least three weekdays and one weekend day. As a result, we obtained the average time spent in different PA intensities (sedentary, light, moderate and vigorous) per hour (min/h).

##### Screen time

Using a 7-d sedentary behaviour diary, parents reported the frequency and duration (hours or minutes) of their child’s screen use separately for (i) watching TV, (ii) watching DVD or videos, (iii) using tablet computers or smartphones and (iv) using computers or playing computer games. The diary was based on a previously validated diary, which was further modified into the Finnish context and has shown to have good reproducibility^(34)^. In the analyses, we used two continuous ST variables: (1) TV and DVD watching time and (2) tablet computer, smartphone and computer time. Both were calculated as a weighted mean: (mean for weekdays × 5 + mean for weekend days × 2)/7 for all children who had diary data from at least three weekdays and one weekend day, and descriptive results are presented in min/d.

##### Sleep

To identify the sleep and wake states and to infer sleep onset, wake-up time and sleep duration, we obtained sleep estimates from the accelerometers by applying a hidden Markov model algorithm. The accelerometer-derived sleep estimates have been described earlier and are more sensitive at recognising associations between sleep and weight status compared with parental reports^(35)^. Sleep duration was defined as the difference between sleep onset and wake-up time (h), and sleep efficiency was calculated as the time spent asleep divided by total sleep duration. A weighted mean (mean for weekdays × 5 + mean for weekend days × 2)/7 was used for both sleep duration and sleep efficiency and calculated for all children who had accelerometer data from at least three weekdays and one weekend day. Three items, based on the Children’s Sleep Habits Questionnaire^(36)^, were used to build a sleep consistency variable, which has been previously used in the DAGIS study sample^(14)^. Parents were asked to recall how many times during the last typical week the child (1) went to bed at the same time at night, (2) slept the appropriate amount and (3) slept approximately the same amount each day. The answer options were recoded as 1 (never), 2 (1–2 times per week), 3 (3–4 times per week), 4 (5–6 times per week) and 5 (daily), and a mean of the three items was calculated to reflect sleep consistency, with higher values indicating greater consistency.

#### Other individual-level factors

##### Temperament

The parents completed the Children’s Behaviour Questionnaire, very short form^(37)^. The questionnaire includes thirty-six items, and response options range from 1 (extremely untrue) to 7 (extremely true). Three broad temperament dimensions, each assessed with twelve items, were derived: surgency, negative affectivity and effortful control. Surgency contains traits, such as impulsivity, high activity level and low shyness, and describes a child’s emotional and motor reactivity, whereas negative affectivity can be interpreted as a child’s general tendency to experience uncomfortable emotions and is characterised by anger, frustration, fear and low soothability^(38)^. Effortful control, in turn, can be defined as the level of self-regulatory capacity and includes, for example, inhibitory control, attentional focusing and perceptual sensitivity^(38)^. For each participant, we calculated a mean score for each temperament dimension, and these scores were used as continuous variables in the analyses. The questionnaire has demonstrated acceptable internal consistency and criterion validity both in earlier studies^(37)^ and within the current sample, in which the Cronbach’s alphas were 0·80 for surgency, 0·76 for negative affectivity and 0·74 for effortful control^(24)^.

##### Hair cortisol concentration

We used HCC to assess long-term stress. Hair samples consisting of approximately forty hairs were collected from the posterior vertex of the scalp by trained preschool personnel and cut as close to the scalp as possible. The hair samples were packed in foil and a small plastic bag and sent to a laboratory for analysis. The laboratory personnel cut the strands into two separate 2-cm segments. Chemiluminescence immunoassay (IBL) was used to measure HCC from the hair samples. The intra- and inter-assay coefficients of variance (CV%) were below 12 % for both. In this article, we use HCC (pg/mg) measured from the first 2-cm hair segment, which roughly indicates the cumulative HCC during the past 2 months. As the distribution of the HCC values was skewed, log_10_ transformation was used in the analyses.

##### Confounding factors

Parents reported the sex and birth date of the participating children, and age at the beginning of the study (in years, continuous) was used in the analyses. In addition, each respondent parent reported their own and their spouse’s educational levels using six predefined response options (comprehensive school, vocational school, secondary school, bachelor’s degree or equivalent, master’s degree, licentiate or doctoral degree), which were further broken down into three categories (secondary school or lower, bachelor’s degree or equivalent, master’s degree or higher). The highest educational level in the family was used as a proxy for the family’s SES.

#### Statistical methods

We used finite mixture models to identify EBRB clusters among the participating children. This latent class analysis method models the distribution of the observed variables and permits the detection of variables relevant for clustering (i.e. variables whose distributions differ between the clusters)^(39)^. Altogether thirty-six EBRB variables (listed in Table 1), including twenty-seven food-, four PA-, three sleep- and two ST-related variables, were used as input in the analysis. Using the R function VarSelCluster in the package VarSelLCM, we ran 1–6-cluster solutions and selected the 2-cluster solution for further inspection based on the Bayesian information criterion (i.e. the 2-cluster solution had the smallest Bayesian information criterion, see Table 2). The algorithm used for variable selection deals with missing values by using the expectation–maximisation algorithm, which assumes missing values to be missing completely at random^(39)^, and the full sample of 864 participating children was thus included in the analysis. The number of participants with missing information was 49 for food consumption variables; 128 for TV and DVD watching time; 139 for tablet computer, smartphone and computer time; 84 for PA variables; 71 for sleep consistency and 136 for sleep duration and sleep efficiency. Altogether 572 participants (66 %) had no missing data, 137 participants (16 %) had one, 81 participants (9 %) two, and 74 participants (9 %) three or more EBRB missing. Each participant was assigned to the cluster that the participant had the highest estimated probability of belonging to. Clusters were labelled according to their most distinctive characteristics.

**Table 2:** BIC values and the number of relevant variables by the number of clusters in the DAGIS study. The lowest BIC value is indicative of the best model (bolded)

Number of clusters	BIC	Number of relevant variables
1	−83 564	36/36
2	**−82 496**	**14/36**
3	−81 991	18/36
4	−81 703	13/36
5	−81 590	12/36
6	−81 607	13/36

BIC, Bayesian information criterion.

We used Student’s t- and Chi-Squared tests to compare the EBRB, age, sex, family SES, temperament dimensions, HCC and weight status between the children categorised in the two clusters. Multilevel logistic regression models with random intercept were used to separately investigate the associations of cluster membership, temperament dimensions, HCC, age, sex and SES (explanatory variables) with overweight (outcome). We also ran a full model: a multilevel logistic regression model with random intercept and with all the explanatory variables entered simultaneously. A multilevel model with three levels (preschool; family; individual children) was used because our recruitment strategy was preschool-based and could have led to clustering of the participants. The middle level (family) was included because the sample included children living in the same household (altogether ninety families had two or three children participating in the study). All independent variables included in the logistic regression models were centred (i.e. for continuous variables, the general mean of the variable was subtracted from the individual values; for binary variables, the categories were set at –0·5 and 0·5; for three-class variables the categories were set at –1, 0 and 1). To estimate the robustness of the results, we used multilevel linear regression models to examine the associations of cluster membership, temperament dimensions, HCC, age, sex and SES (explanatory variables) with BMI-SDS (outcome) as a sensitivity analysis. Additional sensitivity analyses were performed by running multilevel logistic regression models without (1) temperament dimensions and (2) HCC. The analyses included all children with data on the variables in question and were performed using R.

## Results

**Fig. 2:**
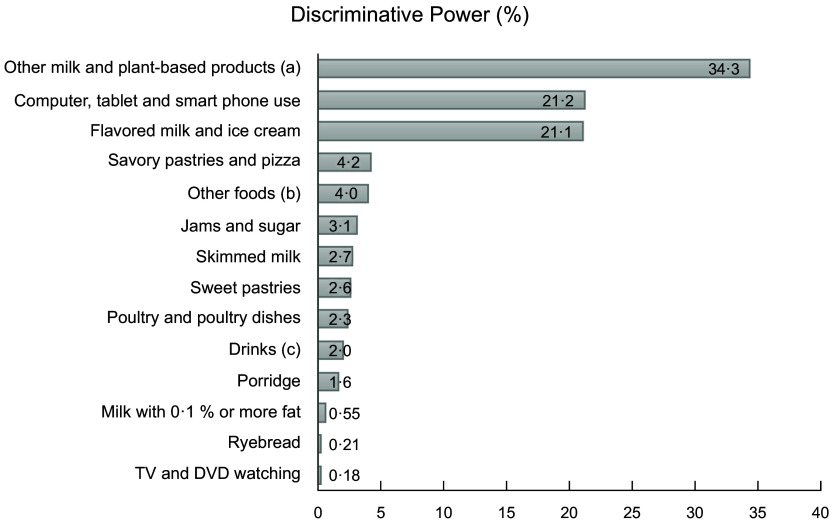
The percentage of discriminative power for the variables relevant for clustering in the cross-sectional DAGIS survey (*n* 864)

Table 2 shows the Bayesian information criterion values and number of relevant variables for the 1–6-cluster solutions. The 2-cluster solution with fourteen variables relevant for clustering had the best fit (the smallest Bayesian information criterion value) and was thus chosen for subsequent analyses. The variables most relevant for clustering were other milk- and plant-based products (cream, sour milk, milk-based desserts and sauces, plant-based drinks and other plant-based alternatives; the percentage of discriminative power 34 %); computer, tablet and smart phone use (21 %) and flavoured milk and ice cream (21 %). Figure 2 illustrates the percentage of discriminative power for the variables relevant for clustering.

Table 1 shows how the two clusters differed regarding the EBRB variables. Of the 864 participants, 304 (35 %) were categorised into cluster 1. The cluster was labelled ‘Unhealthy diet, excessive screen time’ based on the participants’ lower consumption of foods typically considered healthy (e.g. rye bread, porridge, skimmed milk), higher consumption of foods typically considered unhealthy (e.g. flavoured milk and ice cream, savoury pastries and pizza, sweet pastries, drinks) and longer computer, tablet and smart phone usage time. Cluster 2 consisted of 560 (65 %) participants and was labelled ‘Healthy diet, moderate screen time’. The clusters did not differ in terms of PA and sleep variables.

Table 3 shows the descriptive characteristics of the participating children overall and by cluster membership. On average, they were 4·7 years old, and 52 % of them were boys. According to the Finnish cut-offs^(29)^, 16 % of the participating children were with overweight, whereas the respective share was 12 % using the IOTF cut-offs^(30)^. Of the temperament dimensions, participants in the ‘Unhealthy diet, excessive screen time’ cluster scored higher in negative affectivity and lower in effortful control compared with participants in the ‘Healthy diet, moderate screen time’ cluster (3·81 *v*. 3·63, *P* = 0·01; 5·11 *v*. 5·25, *P* = 0·01). In addition, participants in the ‘Unhealthy diet, excessive screen time’ cluster were somewhat older (4·81 years *v*. 4·69 years, *P* = 0·06). Participants in the clusters did not differ in terms of surgency, HCC, sex, SES or weight status.

**Table 3:** Descriptive characteristics of the participating children

	All participants (*n* 864)			Participants in cluster 1* (max *n* 304)			Participants in cluster 2† (max *n* 560)			
Continuous variables	Mean	sd	Missing data (*n*)	Mean	sd	Missing data (*n*)	Mean	sd	Missing data (*n*)	*P* value
Age (years)	4·73	0·90	0	4·81	0·92	0	4·69	0·88	0	0·06
Temperament dimensions										
Surgency	4·69	0·86	113	4·73	0·84	37	4·68	0·87	76	0·46
Negative affectivity	3·70	0·87	113	3·81	0·91	37	3·63	0·84	76	0·01
Effortful control	5·20	0·72	113	5·11	0·72	37	5·25	0·72	76	0·01
Hair cortisol concentration (pg/mg, log_10_-transformed)	1·11	0·67	187	1·13	0·69	62	1·10	0·65	125	0·47
Categorical variables	*n*	%	Missing data (*n*)	*n*	%	Missing data (*n*)	*n*	%	Missing data (*n*)	*P* value
Sex			1			0			1	
Boy	452	52		170	56		282	50		
Girl	411	48		134	44		277	49		0·14
Parental educational level			5			2			3	
Low	201	23		67	22		134	24		
Middle	355	41		138	46		217	39		
High	303	35		97	32		206	37		0·15
Weight status, Finnish cut-offs‡			56			15			41	
Normal weight or thin	682	84		244	84		438	84		
Overweight or obese	126	16		45	16		81	16		1·00
Weight status, IOTF cut-offs§			56			15			41	
Normal weight or thin	712	88		258	89		454	87		
Overweight or obese	96	12		31	11		65	13		0·52

* Unhealthy diet, excessive screen time.

† Healthy diet, moderate screen time.

‡ Cut-offs based on the age- and sex-specific growth reference^(36)^.

§ Cut-offs set by the International Obesity Task Force (IOTF)^(38)^.

In the separate model, a one-unit increase in surgency associated with a 40 % increase in the odds of overweight (OR = 1·40, 95 % CI 1·09, 1·80), and the association strengthened slightly in the full model (OR = 1·63, 95 % CI 1·17, 2·25) (Table 4). EBRB clusters did not associate with the odds of overweight. The odds of overweight were lower in girls than in boys (OR = 0·38, 95 % CI 0·25, 0·58 in the separate model and OR = 0·38, 95 % CI 0·22, 0·66 in the full model). When the IOTF cut-offs were used, increasing age associated with an increase in the odds of overweight in the separate model (OR = 1·47, 95 % CI 1·14, 1·89) (see online supplementary material, Supplemental Table 1). In the full model, both increasing age (OR = 1·40, 95 % CI 1·04, 1·90) and higher scores in surgency (OR = 1·47, 95 % CI 1·03, 2·11) associated with increased odds of overweight.

**Table 4:** Multilevel logistic regression models explaining overweight/obesity according to Finnish cut-offs^(36)^

	Separate models (*n* 642–808)*		Full model† (*n* 564)	
	OR	Lower CI, Upper CI	OR	Lower CI, Upper CI
Cluster membership (ref. cluster 1‡) (*n* 808)	1·00	0·67, 1·50	0·93	0·56, 1·55
Surgency (*n* 706)	**1·40**	**1·09**, **1·80**	**1·63**	**1·17**, **2·25**
Negative affectivity (*n* 706)	0·87	0·69, 1·11	0·98	0·73, 1·32
Effortful control (*n* 706)	0·86	0·65, 1·14	1·13	0·78, 1·65
Hair cortisol (log) (*n* 642)	1·00	0·72, 1·38	0·87	0·59, 1·29
Age (*n* 808)	1·18	0·95, 1·464	1·20	0·92, 1·58
Sex (ref. boy) (*n* 808)	**0·38**	**0·25**, **0·58**	**0·38**	**0·22**, **0·66**
Parental education (ref. low) (*n* 803)	0·98	0·75, 1·26	0·99	0·71, 1·38

Bold values denote statistical significance (p<0.05).

* Sample size for the models is shown in parenthesis after each of the explanatory variables.

† Includes all variables.

‡ Unhealthy diet, excessive screen time.

The sensitivity analyses showed results parallel to the previously presented. Surgency was positively associated with BMI-SDS (model estimate 0·09, 95 % CI 0·01, 0·17) in the separate model and remained close to statistical significance also in the full model (model estimate 0·10, 95 % CI –0·01, 0·20). Unlike in the logistic model, sex was not associated with BMI-SDS in the linear regression model. The association between sex and overweight was similar to those previously presented in full models without (1) temperament dimensions and (2) HCC (ref. boys, OR = 0·39, 95 % CI 0·24, 0·62 in the model without temperament dimensions; OR = 0·40, 95 % CI 0·25, 0·69 in the model without HCC). Similarly, the association between surgency and overweight was similar to the previously presented results in the full model without HCC (OR = 1·41, 95 % CI 1·07, 1·86).

## Discussion

This article identified two distinct EBRB clusters among Finnish preschoolers. The first was labelled ‘Unhealthy diet, excessive screen time’, as it was characterised by lower consumption of foods typically considered healthy and higher consumption of foods typically considered unhealthy along with a longer time spent using computers, tablet computers or smart phones. The second cluster, ‘Healthy diet, moderate screen time’ was, in turn, characterised by a healthier diet and moderate ST. Participants in the ‘Unhealthy diet, excessive screen time’ cluster had higher scores in negative affectivity and lower scores in effortful control than participants in the ‘Healthy diet, moderate screen time’ cluster. Higher scores in surgency associated with increased odds of overweight, whereas cluster membership and HCC did not associate with overweight. To the best of our knowledge, this is the first study to examine EBRB clusters using detailed data on food consumption, PA intensities, screen time and sleep, and taking energy intake into account. Another unique aspect in this study was the simultaneous examination of EBRB clusters, temperament characteristics and HCC with regard to overweight.

Multiple methodological differences between the current and earlier studies somewhat hinder their comparison. Nonetheless, the identified EBRB clusters were rather similar to those found earlier. For example, clustering of dietary behaviours and screen time, PA or sedentariness has been identified in previous studies^(7–12)^. However, neither sleep consistency, sleep duration nor sleep efficiency was relevant for clustering in our study, suggesting that the distributions of these variables were similar between the clusters. Not many studies investigating EBRB clusters among children have included sleep, but two studies have yielded quite consistent results suggesting that healthy sleep behaviours correlate with other healthy EBRB^(7,12)^. However, these studies used parent-reported questionnaires to assess sleep. As mentioned earlier, parents may overestimate preschoolers’ sleep duration^(18)^, and this overestimation is potentially more substantial among the parents of children with healthy dietary and PA habits, which may have affected the results. In a French study, sleep duration additionally included both nap and night-time sleep duration among 2-year-olds^(7)^, whereas we only included night-time sleep, which may explain the differences in EBRB clusters. Furthermore, cultural aspects may impact the distribution of the sleep variables, which in turn may affect the emerging EBRB clusters. For example, a systematic review has noted that sleep duration can vary between countries in the same region^(40)^, and a multi-country study found that children from Northern Europe slept 0·59 h longer than children in Southern Europe^(41)^. More research with objective sleep measurements is needed to further clarify the possible clustering of sleep with other EBRB.

Unlike the majority of studies published earlier^(7,9,11,13)^, we did not find an association between the identified EBRB clusters and overweight. A possible reason for our discordant finding is that we adjusted the food consumption variables with energy intake. They were thus more descriptive of dietary quality than energy content, which obviously has the greatest impact on overweight. However, energy adjustment would be needed to facilitate comparison with the PA behaviours (energy intake usually correlates with PA) and to allow the inspection of dietary quality. Of the previous studies, only Gubbels *et al*. have used an approach similar to ours by calculating energy intake from each food group^(11)^. However, significant methodological differences between the aforementioned study and our current study still remain. First, Gubbels *et al*. used a longitudinal design and found that higher scores in a ‘Sedentary–snacking pattern’ (much like the ‘Unhealthy diet, excessive screen time’ cluster in our study) at 5 years associated with increased odds of overweight at 7 years. As our study was cross-sectional, it is possible that the effects of the EBRB clusters are currently not evident but may become so in the future. Furthermore, parental control over children’s diets decreases as they grow older, which may allow the clusters to become more prominent with regard to weight. Second, the two studies used different dietary assessment methods. Food records assess dietary intake more accurately, whereas assessing energy intake using a FFQ is prone to error^(42)^. As excess energy intake over long periods of time is known to lead to weight gain, future studies should aim to apply a longitudinal setting and to examine dietary quality as part of EBRB clusters by adjusting food consumption variables with energy intake.

In this study, children in the ‘Unhealthy diet, excessive screen time’ cluster scored higher in negative affectivity and lower in effortful control than did children in the ‘Healthy diet, moderate screen time’ cluster. Similar associations have also been found in a cross-sectional analysis of Canadian 3–5-year-olds, where high effortful control associated inversely and high negative affectivity positively with unhealthy dietary intake^(43)^. Using the same sample as in our current study, we have also demonstrated an association between higher effortful control and more frequent vegetable consumption^(24)^. The current study did not find a relationship between surgency and EBRB clusters, but as hypothesised, higher scores in surgency associated with higher odds of overweight. This finding was independent of the cut-offs (Finnish *v*. IOTF) used for overweight, which suggests an association between the variables might exist. Our results are in line with previous studies: for example, in a US study, 5-year-olds high in surgency were more likely to have a higher BMI z score at the age of 10–15 years^(22)^. However, we were unable to demonstrate a link between overweight and negative affectivity or effortful control, which have been established in other studies^(20)^. Unlike many other studies, our models included behavioural risk factors for obesity (i.e. EBRB clusters) and were thus able to account for potential differences in dietary behaviours and PA. Still, the associations between temperament characteristics and overweight are partly conflicting: for example, we have shown that higher surgency links with lower sedentariness and higher moderate to vigorous PA^(44)^, which in turn associates with lower BMI^(45)^. To further clarify these complex associations, more research examining the associations between temperament, EBRB and overweight is needed, preferably in longitudinal settings and using mediation analysis techniques.

We also found that girls were less likely to be with overweight than boys, which parallels with the results of a nationally representative report from Finland^(46)^. This is an unexpected finding considering that we have shown that girls tend to be more sedentary and have less PA than boys^(47)^, whereas energy intake in boys seems to be higher than in girls^(48)^. A higher BMI can, however, be indicative of higher fat-free mass rather than higher body fatness, and thus, these results should be interpreted with caution. More research is needed to identify biological, cultural and other environmental factors that may predispose Finnish boys to overweight. In this study, HCC did not associate with EBRB clusters or overweight. To the best of our knowledge, no previous studies have examined the association between HCC and EBRB clusters, but some evidence, including a study from our own research group, suggests that long-term stress relates to an unhealthy diet among 3–6- and 5–12-year-olds^(26,49)^. However, in our previous study, HCC did not associate with ST in preschoolers^(23)^, which may explain the lack of association between HCC and EBRB clusters. Moreover, our current study was unable to demonstrate a relationship between HCC and overweight, even though a recently conducted meta-analysis found a consistent positive association between HCC and BMI across age groups^(21)^. The lack of association between HCC and overweight in our study may possibly be explained by the smaller number of boys with HCC values (hair samples could not be taken from children with hair shorter than 2 cm, which were mostly boys) and the higher prevalence of overweight among boys. Another explanation may be the choice of variables: we used HCC and BMI-based weight status, whereas the strongest correlations and largest effect sizes have been demonstrated using hair cortisone and waist circumference^(21)^.

The results of this study have several public health implications. First of all, it is crucial to understand the associations between EBRB, as sedentariness coupled with excessive energy intake contributes to childhood obesity, which, later on, can lead to a range of long-term health issues, such as type 2 diabetes and CVD and cause greater economic burden to the society^(50)^. In addition to contributing to energy imbalance, more frequent consumption of snacks and beverages high in sugar, salt and saturated fat together with infrequent consumption of fruits and vegetables can result in deficiencies in the intake of essential nutrients and thus predispose children to compromised growth and development. When the children grow older, excessive ST might also affect their social and emotional development^(51)^. Furthermore, unhealthy EBRB might contribute to poor academic performance^(52)^, which may further associate with deteriorated mental health. Together with previously published results^(20,22)^, this study underlines the role of temperament with regard to child overweight and highlights the complexity regarding the associations between temperament, EBRB and overweight. More challenges are introduced by the modern, abundant and ubiquitous food environments, which may expose children to unhealthy dietary behaviours differently according to their temperamental characteristics^(53)^. Parents’ and early educators’ knowledge of children’s health behaviours and associated temperamental characteristics should be promoted to support children’s healthy EBRB and weight. Addressing these public health implications requires a diverse range of approaches involving parents, preschools, municipalities, healthcare providers, policymakers, as well as the food and entertainment industries.

This study has limitations that need to be accounted for while interpreting the results. First, we used a cross-sectional design, which limits the conclusions that can be made based on the results. For example, even though EBRB clusters did not associate with overweight in this study, it is possible that the association only becomes apparent after years of energy imbalance and unhealthy diet. Thus, longitudinal studies examining the effects of early childhood EBRB on overweight are badly needed. Another limitation is the rather low participation rate (< 30 %), which hampers the representativeness of the results. In terms of SES, the current sample was somewhat biased: ca. 70 % of mothers and over 50 % of fathers had a Bachelor’s level or higher education, whereas approximately 40 % of 35–39-year-old Finnish adults in the general population are as highly educated^(54)^. It is well-known that participants with higher education and favourable health tend to have higher response rates in epidemiological studies^(55)^. Thus, the participants in the current study might have been more health-oriented leading to less variation in EBRB. Nevertheless, the prevalence of overweight or obesity was 15 % in our sample, whereas the respective percentage among 2–6-year-olds in Finland was approximately 23 % in boys and 13 % in girls in 2016^(46)^, suggesting that selection bias in terms of overweight was not considerable.

Despite these limitations, our study has several strengths. The distinctive methodological aspect of our study was our ability to assess both PA and dietary behaviours using more accurate methods (accelerometer and food records) than previous studies, which have mainly utilised questionnaire data. Using food records enabled us to adjust the food consumption variables with energy intake and examine dietary quality within the EBRB clusters. Moreover, rather than relying on theory-driven measures for dietary quality or identifying data-driven dietary patterns before EBRB clustering, we included all food groups and other EBRB in the clustering analysis. This approach allowed each of the food groups to separately establish distinct associations with other EBRB while simultaneously taking the whole diet into account. Another methodological novelty in our study was the inclusion of three sleep variables, two of which were measured objectively using accelerometers. Accelerometers were also used to measure PA intensities and sedentariness. Accelerometers provide more accurate data compared with parental reports, which can significantly overestimate PA and sleep duration^(18)^. Furthermore, our sedentary behaviour diary was able to separate between the electronic screen uses of various screen types. We also acknowledged the possible roles of temperament and HCC with regard to EBRB clusters and overweight. Our analyses included a relatively large sample of preschool-age children, which is an interesting age group from the obesity prevention viewpoint, as prudent EBRB are typically formed in childhood and seem to track into adulthood^(56)^. In addition, as the finite mixture model used deals with missing values by assuming that they are missing completely at random, we were able to assign all participants into one of the two clusters, which maximised the statistical power in the subsequent analyses.

### Conclusions

This study confirms the findings of several earlier studies, which have showed that unhealthy and healthy EBRB cluster already at preschool age. However, unlike some earlier studies, PA and sleep variables were irrelevant for clustering in our study, suggesting that food consumption and ST are more tightly interrelated EBRB. High negative affectivity and low effortful control associated with the unhealthy EBRB cluster. We were unable to observe an association between EBRB clusters and overweight, but children higher in surgency were more likely to be with overweight or obesity. Thus, customised support acknowledging the child’s temperament could be profitable for promoting healthy EBRB and maintaining a healthy weight.

## Supporting information

Vepsäläinen et al. supplementary material Vepsäläinen et al. supplementary material

## Supplementary material

For supplementary material accompanying this paper visit https://doi.org/10.1017/S1368980024000612
