# Supplementary material for: How energy balance-related behaviours, temperament, stress and overweight associate: a cross-sectional study of Finnish preschoolers
Source: Public Health Nutr. 2024 Mar 4;27(1):e93. doi: 10.1017/S1368980024000612 (PMC10966850; doi:10.1017/S1368980024000612)
Supplement: Vepsäläinen et al. supplementary material [file S1368980024000612sup001.docx]

How energy balance-related behaviors, temperament, stress, and overweight associate: A cross-sectional study of Finnish preschoolers

Vepsäläinen H, Korkalo L, Skaffari E, Abdollahi AM, Pajulahti R, Lehto R, Engberg E, Leppänen MH, Lehto E, Ray C, Roos E, Erkkola M.

Supplementary Tables

Supplementary Table 1. Multilevel logistic regression models explaining overweight/obesity according to cut-offs set by the International Obesity Task Force (IOTF) ^(30)^.

|  | Separate models ^a^ (n=642–808) | | |  | Full model ^b^ (n=564) | | |
| --- | --- | --- | --- | --- | --- | --- | --- |
|  | OR | Lower CI | Upper CI |  | OR | Lower CI | Upper CI |
| Cluster membership (ref. cluster 1 ^b^) (n=808) | 1.18 | 0.73 | 1.89 |  | 1.07 | 0.60 | 1.90 |
| Surgency (n=706) | 1.19 | 0.89 | 1.57 |  | **1.47** | **1.03** | **2.11** |
| Negative affectivity (n=706) | 0.85 | 0.64 | 1.13 |  | 0.90 | 0.65 | 1.25 |
| Effortful control (n=706) | 0.98 | 0.71 | 1.37 |  | 0.90 | 0.5~~9~~ | 1.36 |
| Hair cortisol (log) (n=642) | 0.84 | 0.58 | 1.22 |  | 0.90 | 0.58 | 1.38 |
| Age (n=808) | **1.47** | **1.14** | **1.89** |  | **1.40** | **1.04** | **1.90** |
| Sex (ref. boy) (n=808) | 0.97 | 0.62 | 1.52 |  | 1.07 | 0.59 | 1.93 |
| Parental education (ref. low) (n=803) | 0.94 | 0.70 | 1.26 |  | 1.05 | 0.73 | 1.52 |

^a^ Sample size for the models are shown in parenthesis after each of the explanatory variables ^b^ Includes all variables
^c^ Unhealthy diet, excessive screen time
